# Complete and Circularized Genome Sequences of Three *Xanthomonas* Strains Pathogenic on Soybean and Alfalfa

**DOI:** 10.1128/MRA.00537-21

**Published:** 2021-08-05

**Authors:** Mylène Ruh, Martial Briand, Sophie Bonneau, Armelle Darrasse, Marie-Agnès Jacques, Nicolas W. G. Chen

**Affiliations:** a University of Angers, Institut Agro, INRAE, IRHS, SFR QUASAV, Angers, France; University of Arizona

## Abstract

We report the complete and circularized genome sequences of two strains of Xanthomonas citri pv. glycines causing bacterial pustule on soybean and one strain of Xanthomonas euvesicatoria pv. alfalfae causing bacterial leaf and stem spot on alfalfa. These assemblies provide high-quality material for functional and evolutionary studies of these legume pathogens.

## ANNOUNCEMENT

Xanthomonas euvesicatoria pv. alfalfae and Xanthomonas citri pv. glycines are two phylogenetically distant *Xanthomonas* pathovars causing disease on different legume species, including the model plants Medicago truncatula and soybean (Glycine max) (1, 2). Both bacteria are seed transmitted and can cause severe losses under warm and moist conditions (3, 4). However, while the host range of *X. citri* pv. glycines seems restricted to soybean, *X. euvesicatoria* pv. alfalfae can infect different legume species, including its main host alfalfa (*Medicago sativa*), as well as soybean, clover (*Trifolium* spp.), vetch (*Vicia* spp.), and *M. truncatula* (3, 4). Previously, HiSeq Illumina genome sequences were released for *X. euvesicatoria* pv. alfalfae and *X. citri* pv. glycines pathotype strains CFBP 3836 and CFBP 2526, both isolated in Sudan, and *X. citri* pv. glycines strain CFBP 7119, isolated in Brazil (5, 6).

Here, we produced PacBio sequences for the same three strains and performed *de novo* assemblies using both PacBio and HiSeq reads. Bacteria were grown on Trypticase soy (TS) agar for 2 days at 28°C, and then 5 to 6 clones were scraped, pooled, and cultured overnight on 10% TS agar to obtain fresh cultures. A loop (∼5 μl) of cells was then suspended in sterile distilled water and collected by centrifugation. Genomic DNA was extracted using the Wizard genomic DNA purification kit (Promega) according to the manufacturer’s recommendations. DNA was sheared using g-TUBE columns (Covaris). PacBio SMRTbell libraries were prepared from ∼10 μg of genomic DNA, and size selection to 15 to 20 kb was done using BluePippin cassettes (Sage Scientific).

Sequencing was done using the PacBio RS II technology with P5-C3 chemistry at the Icahn School of Medicine at Mount Sinai (New York, NY). One single-molecule real-time (SMRT) cell per strain was used, leading to ∼100,000 reads with an *N*_50_ value of ∼20 kbp, which corresponded to 252× to 308× genome coverage (Table 1). The reads were filtered using PreAssembler Filter v1 of SMRT Portal v2.3 (Pacific Biosciences, Inc., CA) with a minimum subread length of 500, minimum polymerase read quality of 0.80, and minimum polymerase read length of 100 bp. Assembly was performed using Canu v1.5 (7) with the setting genomeSize = 5m. Circularization was done using Berokka v0.2.3 (https://github.com/tseemann/berokka). For each molecule, the sequence start was fixed using Circlator v1.5.1 (8). Polishing was performed using variantCaller v2.2.2 (https://github.com/PacificBiosciences/GenomicConsensus) with the setting --algorithm best. PacBio assemblies were then corrected with Pilon v1.23 (9) with the setting --mindepth 0.5, using the HiSeq Illumina reads produced previously (5, 6). Default parameters were used for all software unless otherwise specified.

**TABLE 1:** Statistics

Species	Pathovar	Strain	SRA no. (HiSeq reads)	No. of HiSeq reads	Coverage (HiSeq) (×)	SRA no. (PacBio reads)	No. of PacBio reads	Coverage (PacBio) (×)	*N*_50_ (bp)	GenBank assembly accession no.	Size (bp)	G+C (%)	No. of plasmids	No. of CDSs
*X. euvesicatoria*	alfalfae	3836	SRR14026743	37,799,928	1,500	SRR14028218	102,443	255	19,326	GCA_017724035.1	5,120,436	64.7	1	4,253
*X. citri*	glycines	2526	SRR14026742	75,568,393	3,000	SRR14028219	97,171	252	19,333	GCA_017723855.1	5,304,469	64.6	1	4,477
*X. citri*	glycines	7119	SRR14027991	47,138,978	1,885	SRR14028187	112,444	308	21,508	GCA_017723895.1	5,581,022	64.4	2	4,743

The genomes consisted of a chromosome plus one plasmid for strains CFBP 3836 and CFBP 2526, while strain CFBP 7119 contained two plasmids. The total genome sizes were 5,120,436 bp, 5,304,469 bp, and 5,581,022 bp for strains CFBP 3836, CFBP 2526, and CFBP 7119, respectively, with respective G+C contents of 64.7%, 64.6%, and 64.4% (Table 1). Using CheckM v1.0.7 (10), the genomes were estimated to be >99.63% complete and <0.73% contaminated.

Coding sequence (CDS) predictions were retrieved from the NCBI Prokaryotic Genome Annotation Pipeline (11). Strains CFBP 3836, CFBP 2526 and CFBP 7119 were predicted to contain 4,253, 4,477, and 4,743 CDSs, respectively (Table 1). The three genome sequences comprised the usual arsenal of genes involved in *Xanthomonas* pathogenicity, including type III effectors, among which were genes encoding transcription activator-like effectors (12). In all, these circularized genomes offer high-quality materials for further comparative studies that could help in understanding the genetic determinants of *Xanthomonas* specificity to different legume species.

### Data availability.

We deposited all HiSeq Illumina reads, PacBio RS II reads, and assemblies at GenBank under the accession numbers listed in Table 1.
